# Self-assessment differences between genders in a low-stakes objective structured clinical examination (OSCE)

**DOI:** 10.1186/s13104-018-3494-3

**Published:** 2018-06-15

**Authors:** Lorenzo Madrazo, Claire B. Lee, Meghan McConnell, Karima Khamisa

**Affiliations:** 1 Faculty of Medicine, University of Ottawa, 451 Smyth Rd, Ottawa, ON K1H 8M5 Canada; 2 Faculty of Medicine, McGill University, 3605 Mountain St, Montreal, QC H3G 2M1 Canada; 3 Department of Innovation in Medical Education, Faculty of Medicine, University of Ottawa, 451 Smyth Road, Rm 2156, Ottawa, ON K1H 8L1 Canada; 4 Division of Hematology, Department of Medicine, Faculty of Medicine, University of Ottawa, Ottawa, Canada; 5 Ottawa Blood Diseases Centre, The Ottawa Hospital (General Campus), Box 201 A, 501 Smyth Road, Ottawa, ON K1H 8L6 Canada

**Keywords:** OSCE, Self-assessment, Undergraduate medical education, Gender, Peer-assisted learning

## Abstract

**Objective:**

Physicians and medical students are generally poor-self assessors. Research suggests that this inaccuracy in self-assessment differs by gender among medical students whereby females underestimate their performance compared to their male counterparts. However, whether this gender difference in self-assessment is observable in low-stakes scenarios remains unclear. Our study’s objective was to determine whether self-assessment differed between male and female medical students when compared to peer-assessment in a low-stakes objective structured clinical examination.

**Results:**

Thirty-three (15 males, 18 females) third-year students participated in a 5-station mock objective structured clinical examination. Trained fourth-year student examiners scored their performance on a 6-point Likert-type global rating scale. Examinees also scored themselves using the same scale. To examine gender differences in medical students’ self-assessment abilities, mean self-assessment global rating scores were compared with peer-assessment global rating scores using an independent samples *t* test. Overall, female students’ self-assessment scores were significantly lower compared to peer-assessment (*p *< 0.001), whereas no significant difference was found between self- and peer-assessment scores for male examinees (*p *= 0.228). This study provides further evidence that underestimation in self-assessment among females is observable even in a low-stakes formative objective structured clinical examination facilitated by fellow medical students.

## Introduction

Accurate self-assessment—the ability to assess one’s own performance globally—is critical to lifelong learning as it allows medical students and physicians to appropriately set goals while identifying strengths and weaknesses [1]. Self-assessment is often measured by the relationship between self-assigned scores and those provided by objective observers where a larger difference in these scores denotes poorer accuracy of self-assessment. Notwithstanding, medical professionals have been shown to have a limited ability to accurately assess their own performance [2, 3].

Given the importance of accurate self-assessments, researchers have examined factors that influence the accuracy of such judgments. Several researchers have argued that self-assessment differs between males and females [4–6]. Specifically, female students tend towards underestimating their performance while male students tend to overestimation [4]. Regarding female students, the tendency to underestimate their performance may be mediated by lower self-confidence [5] and higher anxiety [6]. This association is important because low confidence and high anxiety have been associated with lower self-efficacy—the judgment of one’s ability to perform a certain task successfully, a predictor of student performance [7]. The previous studies cited were carried out in high-stakes settings (i.e. summative objective structured clinical examinations, licensing exams). However, whether gender differences in self-assessment persists in low-stakes—and theoretically less anxiety-inducing—settings is unclear. The objective of this study was to investigate whether female medical students underestimate their self-assessments compared to their male counterparts and compared to their actual performance as determined by peer-assessors in a low-stakes objective structured clinical examination (OSCE).

## Main text

### Methods

#### Study participants

Study participants were recruited from a group of University of Ottawa medical students who volunteered to participate in a mock OSCE (described below). Fourth year medical students were recruited as examiners, third year students as examinees, and first and second year students as standardized patients (SPs). The same examiners remained throughout all three iterations of the mock OSCE. The examinees and SPs each took part in only one iteration. The study was approved by the Ottawa Health Science Network Research Ethics Board. All study participants provided informed consent. Subject identification numbers were assigned in order to anonymize data. Data collected from non-consenting students were discarded and not included in analysis.

#### Study OSCE

The mock OSCE was held at the University of Ottawa medical school and consisted of 5 stations which tested history-taking, physical examination, counselling, and management skills. Cases were based on the specialties represented in the Medical Council of Canada Qualifying Examination (MCCQE) Part II, a high-stakes licensure examination. Each station provided 1 min for students to read the prompt, 7 min to complete the station, and 2 min of feedback from the examiner-totaling 10 min. Cases were written by several medical students and were revised by a faculty member (KK). Peer examiners attended a training session prior to the mock OSCE.

#### Measures

##### Peer-assessment

Fourth-year examiners rated examinees using a station-specific score sheet consisting of a checklist and a 6-point Likert-type global rating scale (GRS), where 1 = inferior and 6 = excellent. The latter was used as a measure of peer-assessment (PA).

##### Self-assessment

To measure self-assessment (SA), examinees were prompted to rank their own performance on a GRS prior to receiving feedback in each station.

#### Data analysis

Two mixed measures analysis of variance (ANOVA) were used to examine the influence of gender (2 levels: male vs. female), assessment format (2 levels: self vs. peer assessment), and station (5 levels: 5 OSCE stations). Gender served as a between-subjects factor, while assessment format and station were within-subject factors. The dependent measures used were the mean GRS score and the mean checklist score. Post-hoc analyses included involved t-tests, all corrected for multiple comparisons using Bonferroni corrections.

### Results

Thirty-three (15 males, 18 females) third-year students were included in the analysis. Participants scored themselves lower than their peers [*F* (1, 31) = 21.04, *p *< 0.001, $\eta_{P}^{2}$ = 0.404]. Furthermore, females marked themselves lower than males [*F* (1, 31) = 9.24, *p *= 0.005, $\eta_{P}^{2}$ = 0.230]. The linear model did not show any significant differences in SA-GRS and PA-GRS between stations [*F* (1, 31) = 0.24, *p *= 0.887, $\eta_{P}^{2}$ = 0.001] and did not show any combined interactions between gender, station type, and SA and PA [*F* (1, 31) = 0.24, *p *= 0.887, $\eta_{P}^{2}$ = 0.001].

**Fig. 1:**
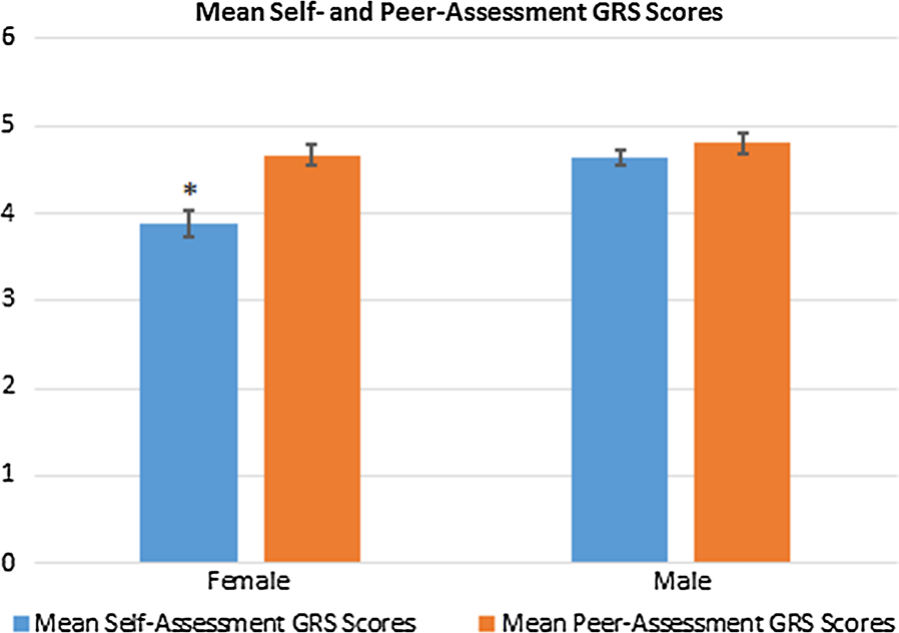
Mean self- and peer-assessment GRS scores stratified by gender. Error bars represent the standard error of the mean. *p < 0.001

As outlined in Fig. 1, females had significantly lower SA-GRS scores compared to PA-GRS scores (3.88 vs. 4.67; *p *< 0.001, *d *= 1.18), whereas no significant difference was found between SA-GRS and PA-GRS scores for male examinees (4.64 vs 4.80; *p *= 0.228, *d *= 0.32). No significant difference existed between male and female students in the achieved checklist (60.32 vs. 56.27; *p* = 0.828) and GRS scores (4.80 vs. 4.67; *p *= 0.452).

### Discussion

Our study demonstrates that underestimation among females is observable even in a low-stakes setting. Notably, despite the disparity in self-assessment between genders, their overall achievement in the mock OSCE did not differ, corroborating the data in the current literature [6]. Our findings—in conjunction with previous research –are noteworthy for several reasons. Firstly, the presence of female underestimation in a low-stakes setting suggests the potential existence of systemic phenomena within medical school that affect mediators such as self-confidence and anxiety among female students. Colbert-Getz et al. [7] found that high anxiety in a high-stakes OSCE contributed to underestimation in performance among female medical students. Even within this low-stakes setting, anxiety may persist due to the pressure from being assessed by fellow medical students [8] or the perceived novelty of the stations. Secondly, these results suggest that similar performance outcomes between male and female students may not necessarily equate to similar perceptions of performance due to variations in anxiety, confidence, and/or self-efficacy [5–7]. Thirdly, socialization within the medical profession may affect male and female trainees differently, potentially contributing to the observed difference in self assessment [9]. Prior research suggests that female medical professionals are more likely to have personal values that are incongruent with institutional values of academic medicine compared to their male counterparts, leading to a reduction in self-confidence and self-efficacy [10]. Whether differences in self-assessment are inherent or acquired upon entry into medical school would be an interesting area of future research.

Curricula should thus move towards recognizing and addressing differences in performance perceptions between genders and promote a more equitable learning experience. A combination of vicarious and personal learning experiences that facilitate the identification of knowledge gaps could help students more accurately appraise their own performance [7].

## Limitations

We acknowledge several limitations in our study. Firstly, our study was restricted to one cohort of medical students in a single institution. Thus, generalizability of these findings may be limited. Secondly, for logistical reasons, we refrained from measuring potential mediators (i.e. self-confidence, anxiety) for self-assessment, preventing us from making definitive conclusions from our results. Thirdly, as we did not instruct examinees to complete the SA-GRS following the feedback, we were not able to see the effect of peer-feedback on the accuracy of SA scores. Future research should explore differences in how male and female students approach and process self-assessment as well as factors that might contribute to this difference. This would better guide teaching and assessment in undergraduate medical curricula.

## Abbreviations

ANOVA: analysis of variance
GRS: global rating scale
MCCQE: Medical Council of Canada Qualifying Examination
OSCE: objective structured clinical examination
PA: peer assessment
SA: self assessment
SP: standardized patient

## References

[1] Eva KW, Regehr G (2005). Self-assessment in the health professions: a reformulation and research agenda. Acad Med.

[2] Gordon MJ (1991). A review of the validity and accuracy of self-assessments in health professions training. Acad Med.

[3] Davis DA, Mazmanian PE, Fordis M, Van Harrison R, Thorpe KE, Perrier L (2006). Accuracy of physician self-assessment compared: a systematic review. JAMA.

[4] Blanch-Hartigan D (2011). Medical students’ self-assessment of performance: results from t hree meta-analyses. Patient Educ Couns.

[5] Blanch DC, Hall JA, Roter DL, Frankel RM (2008). Medical student gender and issues of confidence. Patient Educ Couns.

[6] Colbert-Getz JM, Fleishman C, Jung J, Shilkofski N (2013). How do gender and anxiety affect students’ self-assessment and actual performance on a high-stakes clinical skills examination?. Acad Med.

[7] Mavis B (2001). Self-efficacy and OSCE performance among second year medical students. Adv Health Sci Educ..

[8] Cushing A, Abbott S, Lothian D, Hall A, Westwood OMR (2011). Peer feedback as an aid to learning—what do we want? Feedback. When do we want it? Now!. Med Teach.

[9] Cruess RL, Cruess SR, Boudreau JD, Snell L, Steinert Y (2015). A schematic representation of the professional identity formation and socialization of medical students and residents: a guide for medical educators. Acad Med.

[10] Pololi LH, Civian JT, Brennan RT, Dottolo AT, Krupat E (2013). Experiencing the culture of academic medicine: gender matters, a national study. J Gen Intern Med.

